# Radiomic Nomogram: Pretreatment Evaluation of Local Recurrence in Nasopharyngeal Carcinoma based on MR Imaging

**DOI:** 10.7150/jca.33345

**Published:** 2019-07-10

**Authors:** Lu Zhang, Hongyu Zhou, Dongsheng Gu, Jie Tian, Bin Zhang, Di Dong, Xiaokai Mo, Jing Liu, Xiaoning Luo, Shufang Pei, Yuhao Dong, Wenhui Huang, Qiuyin Chen, Changhong Liang, Zhouyang Lian, Shuixing Zhang

**Affiliations:** 1 Department of Radiology, Guangdong Provincial People's Hospital/ Guangdong Academy of Medical Sciences, Guangzhou, Guangdong, PR China; 2 Institute of Automation, Chinese Academy of Sciences, CAS Key Laboratory of Molecular Imaging, Beijing, PR China; 3 Medical Imaging Center, First Affiliated Hospital of Jinan University, Guangzhou, Guangdong, PR China; 4 Institute of Molecular and Functional Imaging, Jinan University, Guangzhou, Guangdong, PR China; 5 Affiliated Hospital of Guizhou Medical University, Guiyang, Department of Radiology Guiyang, Guizhou, PR China

**Keywords:** Magnetic Resonance Imaging, Nasopharyngeal Carcinoma, Local Recurrence, Radiomic Feature, Nomogram

## Abstract

**Background**: To develop and validate a radiomic nomogram incorporating radiomic features with clinical variables for individual local recurrence risk assessment in nasopharyngeal carcinoma (NPC) patients before initial treatment.

**Methods**: One hundred and forty patients were randomly divided into a training cohort (n = 80) and a validation cohort (n = 60). A total of 970 radiomic features were extracted from pretreatment magnetic resonance (MR) images of NPC patients from May 2007 to December 2013. Univariate and multivariate analyses were used for selecting radiomic features associated with local recurrence, and multivariate analyses was used for building radiomic nomogram.

**Results**: Eight contrast-enhanced T1-weighted (CET1-w) image features and seven T2-weighted (T2-w) image features were selected to build a Cox proportional hazard model in the training cohort, respectively. The radiomic nomogram, which combined radiomic features and multiple clinical variables, had a good evaluation ability (C-index: 0.74 [95% CI: 0.58, 0.85]) in the validation cohort. The radiomic nomogram successfully categorized those patients into low- and high-risk groups with significant differences in the rate of local recurrence-free survival (*P* <0.05).

**Conclusions**: This study demonstrates that MR imaging-based radiomics can be used as an aid tool for the evaluation of local recurrence, in order to develop tailored treatment targeting specific characteristics of individual patients.

## Introduction

Nasopharyngeal carcinoma (NPC) is a rare malignancy in most parts of the world, with incidence rate of approximately 1.1 cases per 100,000 people 1. However, NPC is more common in South-Eastern Asia, particularly in southern China, with an annual incidence rate of 80 cases per 100,000 people 2-6. Approximately 11% of NPC patients develop tumor recurrence at the primary or/and regional site after definitive therapy 7, 8. With the use of intensity-modulated radiotherapy and concurrent chemoradiotherapy, the control of local recurrence has substantially improved 9, 10. However, only a portion of recurrent NPC patients have benefited from the improved treatment, which may be related to the specific characteristics of individuals 1, 9. The outcome of salvage treatment remains very poor 11, 12, with the survival after local recurrence equating to <18.3 months, and its management remains a difficult issue for clinicians 13, 14. It is therefore vital to define the reliable prognostic factors that could identify NPC patients at low- or high-risk of local recurrence.

Currently, magnetic resonance imaging (MRI) is a traditional and important tool for pretreatment staging and the determination of treatment programs for NPC 15, 16. The traditional MRI, such as T1-weighted (T1-w) imaging and T2-weighted (T2-w) imaging, is mainly based on the anatomy of tumor invasion and does not consider intratumor heterogeneity so that they failed to evaluate the risk of individual patient based on specific intratumor characteristics 17, 18. Many functional MRI sequences have been reported, such as diffusion-weighted imaging (DWI), diffusion tensor imaging (DTI), perfusion-weighted imaging (PWI), intravoxel incoherent motion (IVIM), could reflect intratumor features in different cancers 19, 20. However, they are not as regular sequences for NPC patients, because the inherent anatomic constraints could cause all kinds of artifacts. Recent studies have found that radiomics analysis, which is based on imaging data, could provide additional information reflecting the underlying intratumor heterogeneity 21, 22.

Radiomics, a rapidly emerging field, transforms medical images into mineable high-dimensional quantitative features via a large number of automatically extracted data-characterization algorithms 23, 24. These numerous quantitative imaging features are extracted from entire tumors in different modalities (e.g. computed tomography [CT], MRI, or positron emission tomography-computed tomography [PET/CT]), and they may reflect intratumor heterogeneity which are closely associated with cancer staging, prognostic prediction, and response to treatment 25-27. Identification of the intratumor heterogeneity of individual patients has the potential to yield important insights for targeted therapeutic selection and drug development 22, 28. Several studies have observed the predictive ability of radiomic features in many cancers (e.g. head and neck squamous cell carcinoma 29, lung cancer 30, rectal cancer 31, 32, and breast cancer 33), and revealed that radiomic features are associated with progression-free survival (PFS), recurrence, metastasis, and other clinical outcomes 34, 35. Moreover, an intratumor heterogeneity survey for NPC patients has been assessed with regard to progression-free survival and treatment response 36-38. These previous studies have indicated that radiomic features are an independent factor for the prediction of progression-free survival in advanced NPC patients. However, how radiomic features might differ between patients with different risk of local recurrence has not been established.

The aim of this study was to investigate whether radiomic features were associated with local recurrent NPC. Furthermore, we integrated radiomic features with clinical variables to build a nomogram for stratifying low- or high-risk local recurrent NPC patients.

## Materials and Methods

### Patients

This retrospective study was approved by the ethics committee of Guangdong General Hospital review board. All patients provided written informed consent. Our hospital database was searched to identify all patients with newly diagnosed NPC between May 2007 and December 2014 who were treated with a radiotherapy or chemoradiotherapy regimen. The study eligibility criteria were (a) patients with histologically-confirmed NPC; (b) patients who had not received treatment (surgery, radiotherapy, or chemoradiotherapy) before MRI scans; (c) patients who had undergone pre-treatment MRI scans for review (including CET1-w and T2-w images); and (d) patients who were followed-up every 1-3 months during the first 2 years, every 6 months in years 2-5, and annually thereafter. Patients with known magnetic resonance contraindications, insufficient follow-up data, any treatments (radiotherapy, chemotherapy, or chemoradiotherapy) before their first MRI scan, or a history of previous or synchronous malignant tumors, were excluded. The histological subtype of the patients' tumors was categorized according to the World Health Organization standards and included type I (differentiated keratinizing carcinoma), type II (differentiated non-keratinizing carcinoma), and type III (undifferentiated non-keratinizing carcinoma) 39. Eligible patients were randomly divided into two cohorts at a ratio of 4:3. Eighty patients were assigned to the training cohort, while the remaining 60 patients were assigned to the independent validation cohort.

Baseline clinical variables were collected, including age, gender, T stage, N stage, histology, hemoglobin (HGB), and platelet count 40. The primary endpoint of this study was local recurrence-free survival (LRFS), which was defined as the time from the start of the MR examination until the date of local recurrence 12, 13. The minimum follow-up time for patients without local recurrence was 36 months after the first MR examination. All local recurrences were diagnosed by flexible nasopharyngoscopy and biopsy and/or MRI scanning of the nasopharyngeal area and neck.

### Treatment

The targets of radiation therapy (RT) are the nasopharynx and adjacent at-risk tissues, and both sides of the neck (levels Ib-V, and the retropharyngeal nodes) 1. Patients received a cumulative radiation dose equivalent to 66 Gy or greater to the primary tumor, 60-66 Gy to the involved neck area, and 50 Gy or greater to the adjacent at-risk tissues 41. Early stage patients (stage I-II) were treated with RT alone, while advanced stages (stage III-IV) were treated with RT and concurrent chemotherapy 26, 40. The concurrent chemoradiotherapy regimen comprised of cisplatin (40 mg/m² 5 days per week, 6-7 cycles), beginning on the first day of radiotherapy.

### Radiomics workflow

**Figure 1:**
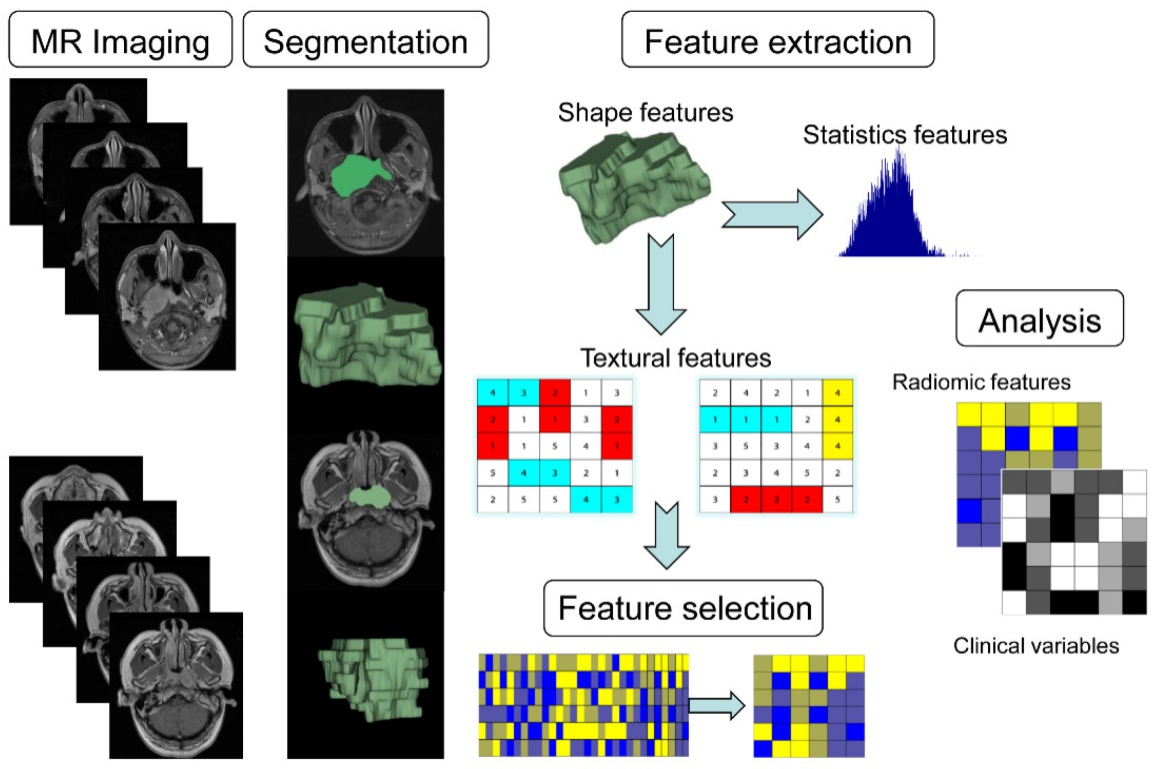
Workflow of the radiomics analysis. (1) Acquisition of high-quality magnetic resonance images. (2) Segmented region of interest (ROI) that contains either the whole tumor rendered in three dimensions. (3) Quantitative features were extracted. (4) Robust features were selected. (5) A combined model was built that integrated the radiomic features and multiple clinical variables.

The radiomics workflow is presented in Fig. 1, including (1) image acquisition, (2) image segmentation, (3) feature extraction, (4) feature selection, and (5) model building.

### Image acquisition, image segmentation, and feature extraction

All patients underwent non-contrast and contrast-enhanced nasopharyngeal and neck 1.5 T MRI scans (Signa EXCITE HD, TwinSpeed, GE Healthcare, Milwaukee, WI, USA). The acquisition parameters were as follows: axial T2-weighted spin-echo images (repetition time [TR]/echo time [TE]: 5000/85 ms, field of view [FOV] = 23 × 23 cm, number of excitations [NEX] = 2.0, slice thickness = 4 mm, spacing between slices = 1.0 mm) and axial contrast- enhanced T1-weighted spin-echo images (TR/TE: 687/16 ms, FOV = 23 × 23 cm, NEX = 2.0, slice thickness = 4 mm, spacing between slices = 1.0 mm). We included axial T2-w Digital Imaging and Communications in Medicine (DICOM) images and CET1-w DICOM images that had been archived using Picture Archiving and Communication Systems (PACS).

The segmentation for region-of-interest (ROI) settings was performed using ITK-SNAP software (open source software; https://itk.org/) All manual segmentations of the tumor were performed by two radiologist with 11 years of experience in head and neck MR image (O.Y. and B.G) in a blinded fashion, and each segmentation was validated by a senior radiologist with 15 years of experience in head and neck MR image (S.Z.). The ROI included the whole tumor and was delineated on both the axial T2-w and CET1-w images in each slice.

The feature extraction was performed in MATLAB R2014a (Mathworks, Natick, MA, USA) using our in-house algorithms designed to extract four different feature classes, including first-order statistics features, shape- and size-based features, statistics-based textural features, and wavelet features, which are described in detail in Supplementary Methods 1. DICOM files (MR images + tumor contours) were imported into the algorithms to extract the radiomic features. A total of 970 complete radiomic features were extracted from MR images, including 485 features from the CET1-w images and 485 features from the T2-w images.

### Feature selection and model building

The recursive feature elimination with logistic regression algorithm (LR-RFE) was used to select the most predictive features with the highest area under the curve (AUC) (containing the first n features) 42, 43. The RFE method continuously eliminates the features that did not significantly contribute to the model based on the iterative method, finally obtaining ranked features based on the number of iterations when the feature was discarded. With the ranked features, different feature sets could be obtained by selecting the top-n features from the ordered sequence (1≤n≤N). The set of first n features was fed into the local recurrence classifier, and its performance for differentiating low- and high-risk of local recurrence in NPC could be evaluated by a receiver-operating characteristic (ROC) curve and AUC. Finally, a subset with the highest AUC (containing the first n features) was selected as the best feature subset for the discrimination task. The LR-RFE method was conducted in the training cohort and validated in the independent cohort. The LR-RFE algorithm was implemented in Python with the Sklearn package. After feature selection, we used Cox regression to build a model. The non-zero coefficient of the selected feature was defined as the radiomic score (Rad-score); the Rad-score was calculated for each patient using a formula derived from the selection features weighted by their regression coefficient. Radiomic features were built using the Rad-score. In this study, the CET1-w and T2-w images were used to establish the prediction model, and obtained the risk scores Rad-score1 and Rad-score2, respectively. Finally, we used cox regression analysis to determine the association of Rad-score_1_, Rad-score_2_, clinical variables with local recurrence in the training cohort. A Rad-score was calculated for each patient derived from identified predictors weighted by their regression coefficient. We calculated the median Rad-score, which classified NPC patients into low-risk and high-risk groups.

### Validation of the radiomic nomogram

The radiomic nomogram, which integrated the radiomic features and clinical variables, was built for prediction based on the Cox proportional hazards regression model. The radiomic nomogram performance was measured quantitatively using the concordance index (C-index) 44. The concordance index and calibration curve were obtained from multivariable Cox proportional hazard regression analyses. The threshold of p value was 0.05 (P < 0.05) for the “good” calibration in this analysis. Patients were stratified into high- or low-risk groups based on the Rad-score, the threshold of which was delimited by using the median Rad-score. Patients with median scores were placed in high-risk groups. We also used a survival analysis to measure the difference in the LRFS between high- and low- risk groups, which was assessed in the training cohort, and then confirmed in the validation cohort 45. We also performed a cox regression analysis to test the significant of clinical variables alone, included age, gender, T stage, N stage, HGB, and platelet count.

### Statistical analysis

The statistical analyses were conducted using R software, version 3.1.3 (http://www.R-project.org). We performed univariate and multivariate analyses in this study. The Kaplan-Meier and log-rank methods were used to analyze the univariate discrimination of the LRFS, which was grouped by models. Survival analysis and Kaplan-Meier survival curves were processed with the “survival” and “survcomp” packages. Multivariate logistic regression was conducted with the “rms” package. Cox proportional hazards regression, nomograms, and calibration curves were calculated by the rms package. C-index calculation was performed with the “Hmisc” package. The clinical characteristics of training and validation cohorts were compared by using an independent samples t-test, χ2 test, or Mann-Whitney U test, as appropriate. We performed correlation analysis to evaluate relationship between clinical variables and local recurrence with the “stats” package. All statistical tests were two-sided, and *P* values of <0.05 were considered significant.

## Results

### Clinical characteristics

A total of 140 patients with pathologically confirmed NPC were included in this study. The training and validation cohorts were similar in terms of baseline clinical variables (*P* >0.05). The median LRFS time was 26 months (range: 3-68 months). The results of correlation analysis showed that N-stage is significant associated with local recurrence but other clinical variables are not significant. The patient characteristics are summarized in Table 1.

### Radiomic feature extraction, feature selection, and model building

In total, the eight features selected from CET1-w images were as follows: CET1-w_1_GLCM_cluster_shade, CET1-w_3_fos_median, CET1-w_3_fos_mean, CET1-w_4_fos_skewness, CET1-w_Surface_to_volume_ratio, CET1-w_6_fos_skewness, CET1-w_4_fos_mean, and CET1-w_4_fos_median. We used these features to establish a Cox proportional hazard model (CET1-w C-index 0.60 [95% CI: 0.49-0.72]. Based on the coefficient of eight features, we calculated the Rad-score1 for CET1-w Cox (Supplementary Methods 3). Similarly, we selected seven features from the T2-w images as follows: T2-w_4_GLCM_cluster_shade, T2-w_6_GLCM_autocorrelation, T2-w_6_GLCM_IMC1, T2-w_1_GLCM_cluster_shade, T2-w_7_fos_mean, T2-w_1_GLRLM_LRHGLE, and T2-w_7_GLCM_sum_average, and established a Cox proportional hazard model (T2-w C-index 0.62 [95% CI: 0.52, 0.71]). The Rad-score2 of T2-w Cox was also calculated (Supplementary Methods 3).

Cox regression analysis determined that Rad-score_1_, Rad-score_2_, gender, age, HGB, and N-stage were associated with local recurrence in the training cohort. A Rad-score was calculated for each patient using the following formula derived from the above independent predictors weighted by their regression coefficient:

Rad-score** =** 0.88663*Rad-score_1_+ 0.50748* Rad-score_2_ + 0.02159*gender+ 0.02012*age + 0.00691*HGB -0.21954*N-stage

**Figure 2:**
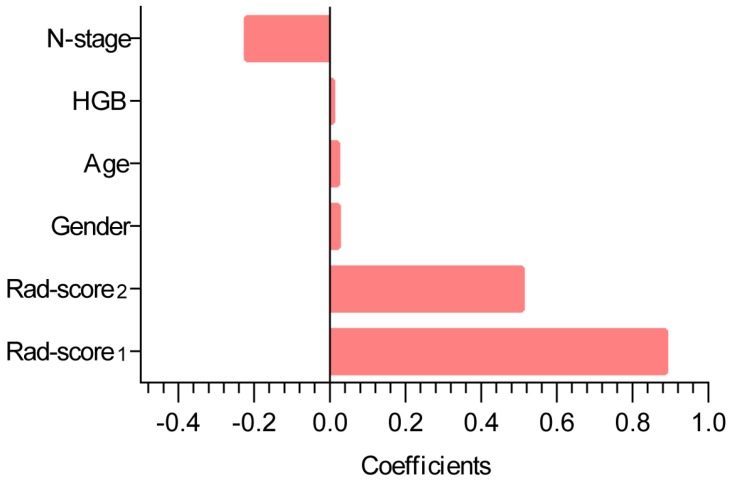
Histogram illustrating the role of the selected parameters that contributed to the radiomic nomogram. The selected parameters are plotted on the y-axis with their coefficients in the Cox analysis plotted on the x-axis.

**Figure 3:**
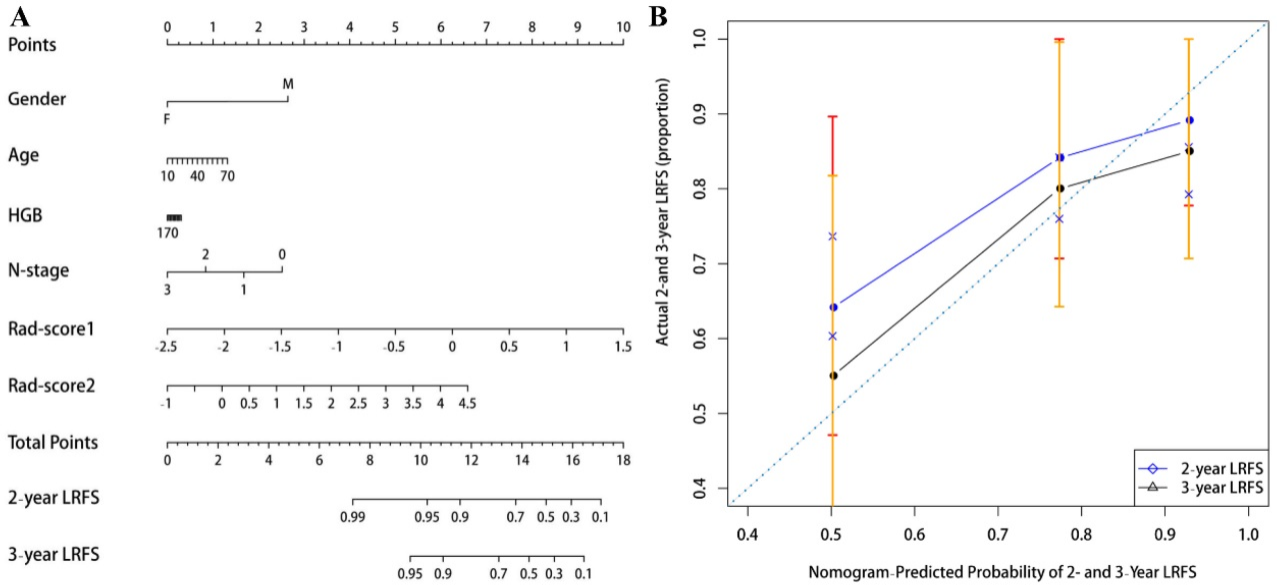
(A) A radiomic nomogram incorporated the Rad-score1, Rad-score2_,_ and clinical variables in the training cohort. (B) Calibration curve of local recurrence free survival probabilities at 2-years (blue line) and 3-years (black line) in nasopharyngeal carcinoma patients. The diagonal dotted line represents an ideal evaluation, while the blue and black solid lines represent the performance of the radiomic nomogram. The closer the fit to the diagonal dotted line, the better the evaluation.

The contribution of the selected parameters with their coefficients for the predictive model construction is presented in the form of a histogram in Fig. 2. The model that incorporated the above independent predictors was developed and presented as the radiomic nomogram (Fig. 3A) and provided a C-index of 0.69 (95% CI: 0.59-0.77). The calibration curves of the radiomic nomogram for the probability of local recurrence occurred at 2 or 3 years, as illustrated in Fig. 3B, and they exhibited better agreement between the estimation with the radiomic nomogram and actual observation.

### Validation of the radiomic nomogram

Using the second radiomic features to similarly extract and select 60 patients as the internal validation cohort, the radiomic nomogram yielded a C-index of 0.74 (95% CI: 0.58-0.85). Good calibration was observed for the probability of local recurrence in the validation cohort (Figure not provided). We also built a clinical model based on the clinical variables. After cox regression analysis, only N stage was remained as independent predictors of local recurrence. The clinical model yielded a C-index of 0.56 (95% CI, 0.66-0.45) in the training cohort and 0.59 (95% CI, 0.68-0.49) in the validation cohort. The radiomic nomogram exhibited better prediction performance than the radiomic features and clinical variables alone, in both the training cohort and the validation cohort.

**Figure 4:**
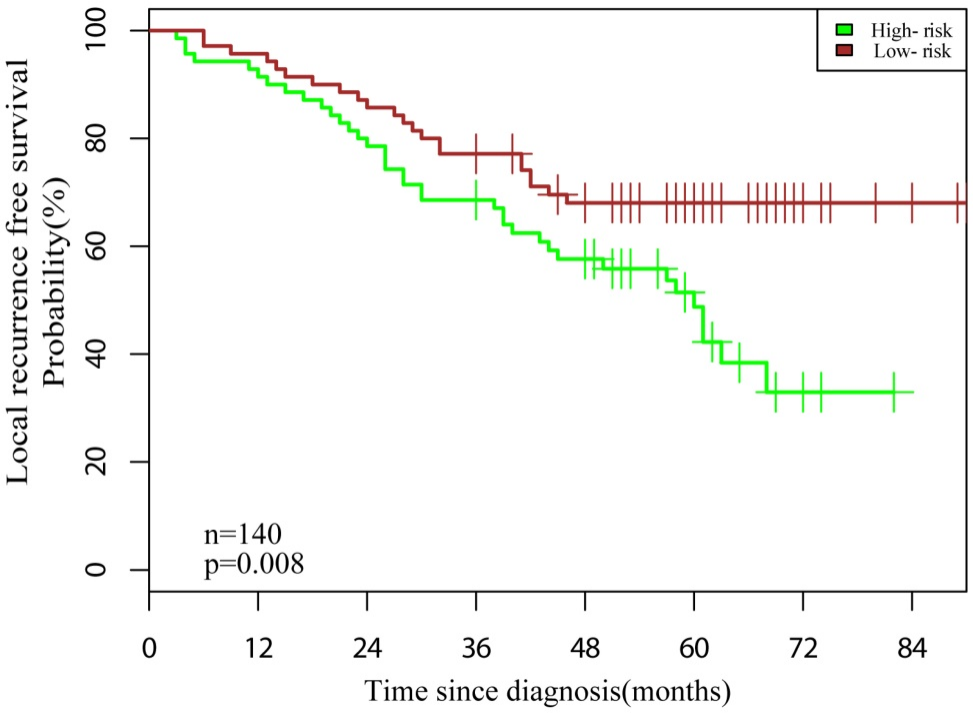
Graphs indicating the results of the Kaplan-Meier survival analyses according to the radiomic features to predict local recurrence-free probability in all datasets. A significant local recurrence free survival difference was noted between patients in the low- and high-risk groups (dataset, n = 140, log-rank test, *P* = 0.008).

The patients were categorized into low- and high-risk groups based on the median Rad-score. Patients in the low-risk group (Rad-score <5.50) were significant longer LRFS than did patients in the high-risk group (Rad-score ≥5.50) (Fig. 4, *P* = 0.008).

## Discussion

Using radiomics-based feature analysis, we found that radiomic features were closely association with local recurrence in NPC patients. In this study, we built a radiomic nomogram that combined the radiomic features and clinical variables to evaluate the risk of local recurrence in NPC patients before initial treatment, and classified NPC patients into high risk and low risk groups. The radiomic nomogram provides a visual tool for optimal clinical decisions, enabling clinicians to perform inexpensive, earlier identification of NPC patients at high risk of local recurrence.

Previous models of NPC demonstrated that MRI-based radiomic features were predictive in progression-free survival and treatment response without offering an evaluation of local recurrence 36, 46. In this study, we found that radiomic features had a closely association with local recurrence in NPC patients, and the predictive performance of the nomogram model underwent significant improvement after the addition of radiomic features. The results can be explained by the fact that the quantitative image features were extracted from entire tumors, and hence they are likely to reflect the intra-tumor heterogeneity. Kwan et al 47 and Vallieres et al 48 have also supported that radiomic features was associated with distant metastasis and could reflect the characteristics of intratumor heterogeneity. The result also demonstrated that the contribution of the CET1-w images to the nomogram was slightly greater than that of the T2-w images. The result may indicate that the T2-w images mainly reflected tumor density and obscure boundaries, while the CET1-w images reflected intra-tumor heterogeneity and architecture (e.g., tumor angiogenesis) 49. This previous finding of angiogenesis associated with tumor invasion lends credence to the possibility of CET1-w image differences possibly reflecting angiogenesis 41.

Age, gender, T-stage, N-stage, hemoglobin, platelet counts have been identified and evaluated in previous studies 50, 51. In this study, after multivariate analyses, age, gender, N-stage, HGB were remained and combined with radiomic features to build a model for the evaluation of local recurrence in NPC patients. Our results indicated that N-stage was negatively associated with local recurrence in NPC patients, while previous findings supported that N-stage was positive association with distant metastasis in NPC patients 52. To validate the relationship between N-stage and local recurrence, we conducted a correlation analysis and the result indicated that N-stage was significantly associated with local recurrence. The possible reasons underlying the negative correlation of N-stage are as follows: first, N-stage was based on the site of adenopathy and that of the greatest diameter 1. However, occult micrometastasis in lymph nodes could exist regardless of the size 53. Second, the irradiation area of the neck is unfixed compared with other head and neck irradiation area by using a thermoplastic head and shoulder mask. Therefore, the cervical lymph node could not receive enough dosing, which may result in local recurrence. Finally, we kept N-stage as an important factor in the nomogram. In addition, age, gender and HGB were remained after filtration. Although these clinical variables provided slightly predictive strength to improve reclassification performance in this study, existing studies have demonstrated that they could serve as important markers of prognosis in NPC patients; therefore, we kept them as candidate factors in the nomogram 10, 50, 54, 55.

Several previous studies have made great efforts to build nomograms for individual local recurrence risk assessment in NPC patients based on clinical variables, such as Chen et al 56, which included age, the neutrophil/leukocyte ratio (NWR), pathological type, primary gross tumor volume, maxillary sinus invasion, ethmoidal sinus, invasion and lacerated foramen invasion. Wang et al 57 also reported that cervical lymph nodes (≥3 cm) were a risk factor for local recurrence in NPC patients treated with intensity-modulated radiotherapy. However, new biomarker that could reflect intratumor heterogeneity still need to be found to guide individual treatment for patients. The radiomic nomogram we developed in this study combined the clinical variables and the specific radiomic features, which selected from first-order statistics, shape descriptors, and texture features, could reflect intratumor heterogeneity. Our results also showed that the radiomic nomogram provides better predictive performance compared with radiomic features and clinical variables alone, as it refers to the combination between multiple clinical manifestations and intratumor heterogeneity. In additional, the radiomic nomogram could serve as both a scoring system and a visualized prediction tool, which could help physicians rapidly evaluate a patient with his/her expected LRFS via a simple calculation in clinical practice. Quantifying both of these risks is critical to the clinical decision of whether patients should receive any specific therapy and, if so, whether it should be less or more “intense.” Notedly, we still need further prospective clinical validation before this model was used for clinic.

There were some limitations in the present analysis. First, this study was performed in an endemic area, single hospital and lacked external validation. Multicenter prospective validation in both NPC-endemic and nonendemic areas with a larger sample size is needed to acquire powerful evidence for clinical application. Second, the algorithms of radiomic model and statistical analysis were relatively unfamiliar and complicated for clinic. There will be a website or application to solve this problem, which doctors could get the results by uploading images and clinical variables.

In conclusion, we developed a radiomic nomogram integrated radiomic features and multiple clinical variables to predict and evaluate local recurrence in NPC patients. The radiomic nomogram classified NPC patients into high-risk and low-risk groups with significant difference in local recurrence-free survival, and it served as a visual tool to identify high-risk individuals who would benefit from aggressive therapeutic strategies.

## Supplementary Material

Supplementary methods.

## Figures and Tables

**Table 1:** Demographic and clinical characteristics of patients in the training cohort and validation cohort. Statistical comparison between the training cohort and the validation cohort was conducted with the Mann-Whitney U test for continuous variables and the χ2 test for categorical variables.

Characteristic	Type	Training Cohort (%) n=80	Validation cohort (%) n=60	P-value
Gender	Male	54 (67.5)	49 (81.7)	0.06
	Female	26 (32.5)	11 (18.3)	
Age (years)	Range	14-71	14-67	0.07
	Median±STD	46±10	42±12	
Overall stage	Ⅰ	1 (1.3)	0 (0)	0.16
	Ⅱ	12 (15)	2(3.3)	
	Ⅲ	46(57.5)	42 (70)	
	Ⅳ	21 (26.2)	16 (26.7)	
T stage	Ⅰ	6 (7.5)	5 (8.3)	0.08
	Ⅱ	30 (37.5)	11 (18.3)	
	Ⅲ	30 (37.5)	33 (55)	
	Ⅳ	14 (17.5)	11 (18.3)	
N stage	Ⅰ	6 (7.5)	5 (8.3)	0.16
	Ⅱ	31 (38.7)	13 (21.7)	
	Ⅲ	36 (45)	37 (61.7)	
	Ⅳ	7 (8.8)	5 (8.3)	
Histology	WHO type I	0 (0)	0 (0)	0.73
	WHO type II	2 (2.5)	1 (1.7)	
	WHO type III	78 (97.5)	59 (98.3)	
Hemoglobin (g/L)	< 136	44 (55)	30 (50)	0.56
	≥ 136	36 (45)	30 (50)	
Platelet counts, ×10^9^/L	< 238	47 (58.8)	26 (43.3)	0.07
	≥ 238	33 (41.2)	34 (56.7)	

## Abbreviations

NPC: Nasopharyngeal carcinoma
MRI: Magnetic resonance imaging
CET1-w: Contrast-enhanced T1-weighted image
T2-w: T2-weighted image
HGB: Hemoglobin
DICOM: Digital Imaging and Communications in Medicine
PACS: Picture Archiving and Communication Systems
ROI: Region-of-interest
AUC: Area under the curve
ROC: Receiver-operating characteristic
