# Supplementary material for: Radiomic Nomogram: Pretreatment Evaluation of Local Recurrence in Nasopharyngeal Carcinoma based on MR Imaging
Source: J Cancer. 2019 Jul 10;10(18):4217–25. doi: 10.7150/jca.33345 (PMC6691694; doi:10.7150/jca.33345)
Supplement: Supplementary file 1 — Supplementary methods. [file jcav10p4217s1.pdf]

Supplementary Information

1. Methods 1：Radiomic feature extraction and selection
2. Methods 2：Radiomic score (Rad-score) calculation formula

Methods 1: **Radiomic feature extraction and selection**

Feature extraction

In our study, 970 radiomic features were extracted including 485 features from the contrast-enhanced T1-weighted (CET1-w) serial MR images and 485 features from the T2-weighted (T2-w) serial MR images. MatLab 2014a software (MathWorks, Natick, MA, USA) was used for feature extraction in the MR images. The 485 features were divided into four groups:

First-order statistics features: To analyze the spatial knowledge of the pixel hue matrix and extract the statistical features of the image, a fuzzy similitude matrix was defined to describe the image feature space. A total of 17 features exist in space including Energy, Entropy, Skewness, Kurtosis, Mean, Maximum value, Minimum value of the image pixel, etc.

Shape- and size-based features: For the original three-dimensional MR image and three-dimensional region of interest (ROI), their shape and size characteristics are typically measured by surface area, volume, surface to volume ratio, maximum three-dimensional diameter, sphericity, spherical disproportion, compactness 1 and 2, for a total of eight features.

Statistics-based textural features: Textural features refer to the visual image that reflects the characteristics of homogeneous phenomena, and reflects the surface properties of the arrangement in the surface structure of the tissues, which change slowly or periodically. Our textural features mainly consisted of the gray-level co-occurrence matrix (GLCM) and the gray-level run-length texture matrix (GLRLM). The GLCM is a pixel matrix function of the distance and angle, which quantifies the correlation by calculating a certain distance and a certain direction between two gray matrices; in this manner, the matrix reflects the integrated information in the direction, interval, amplitude, and frequency [1]. The GLRLM quantifies gray level runs in an image [2, 3]. A gray level run is defined as the length number of continuous pixels that have the same gray level value. We extracted 22 features from the GLCM and 14 features from the GLRLM [4]. The radiomic features in the GLCM mainly comprised energy, entropy, correlation, contrast, homogeneity, autocorrelation, mean, variance, dissimilarity, and angular second moment. The radiomic features in the GLRLM

mainly comprised run length non-uniformity, short/long run emphasis, and gray level non-uniformity.

Wavelet features: In our study, the undecimated three-dimensional wavelet transform was used to decompose the original image. Consider L and H to be low-pass and high-pass functions, X to be the decomposing image, and the wavelet decompositions of X to be labeled as $X_{LLL}, X_{LLH}, X_{LHL}, X_{LHH}, X_{HLL}, X_{HLH}, X_{HHL}, X_{HHH}$. We can obtain eight new images that are decomposed in three directions (x, y, z), where the size of each decomposition is equal to that of the original image and each decomposition is shift invariant. For each decomposition, we computed the first-order statistics and textural features described above. This resulted in 424 features. Ultimately, we extracted 485 features from each of the series.

In conclusion, 970 radiomic features were extracted from the MR images in our study.

Feature selection

In this paper, we selected the patients who experienced recurrence within 1 year and the patients who did not experience any recurrence for >5 years, as a 0/1 label for the selection of the features. We used the recursive feature elimination with logistic regression algorithm (LR-RFE) to select the features, and determined the features of the model based on the highest AUC value of the classification results [5, 6]. From the CETI-w images, we selected eight features as follows: CET1-w_3_fos_median, CET1-w_Surface_to_volume_ratio, CET1-w_3_fos_mean, CET1-w_4_fos_mean, CET1-w_4_fos_median, CET1-w_4_fos_skewness, CET1-w_6_fos_skewness, and CET1-w_1_GLCM_cluster_shade. Similarly, we selected T2-w_7_fos_mean, T2-w_1_GLCM_cluster_shade, T2-w_4_GLCM_cluster_shade, T2-w_6_GLCM_autocorrelation, T2-w_6_GLCM_IMC1, T2-w_7_GLCM_sum_average, and T2-w_1_GLRLM_LRHGLE from the T2-w images.

As demonstrated, our features were all from images that were decomposed by the undecimated three-dimensional wavelet transform. Numbers from ‘1’ to ‘8’ were used to mark the different respective forms of the wavelet transform. The eight features of the CET1-w images were composed of X_{LLL} (marked by “1”), X_{LHL} (marked by “3”),

X_{LHH} (marked by "4"), and X_{HLH} (marked by "6") images. Similarly, the seven features of the T2-w images were composed of X_{LLL} (marked by "1"), X_{LHH} (marked by "4"), X_{HLH} (marked by "6"), and X_{HHL} (marked by "7") images.

In the later discussion, we let X reflect the three-dimensional image matrix with N voxels used to analyze the first-order statistics features and the shape- and size-based features that were selected by the LR-RFE algorithm. Meanwhile, we consider the GLCM and GLRLM to be a matrix size $N_g \times N_g$, defined as $P(i,j;\delta,\alpha)$. Here, the (i,j) element represents the number of times the combination of intensity levels occurs in two pixels in the images that are separated by a distance of δ pixels in direction α, and N_g is the number of discrete gray level intensities, and N_r is the number of different run lengths. In addition, let $p_x(i)=\sum_{j=1}^{N_g}P(i,j)$ $p_y(i)=\sum_{i=1}^{N_g}P(i,j)$ separately refer to the marginal row and column probabilities. Their detailed explanation is as follows:

CET1-w_3_fos_median: The first-order statistics feature that describes the median value of the intensity levels in CET1-w images in X_{LHL} images.

CET1-w_Surface_to_volume_ratio:

$$\text{surface to volume ratio} = \frac{A}{V}$$

CET1-w_3_fos_mean: The first-order statistics feature that describes the mean value of the intensity levels in CET1-w images in X_{LHL} images.

$$mean = \frac{1}{N}\sum_{i}^{N} X(i)$$

CET1-w_4_fos_mean: The first-order statistics feature that describes the mean value of the intensity levels in CET1-w images in X_{LHH} images. The formula is the same as has been shown for ***CET1-w_4_fos_mean.***

CET1-w_4_fos_median: The first-order statistics feature that describes the median value of the intensity levels in CET1-w images in X_{LHL} images.

CET1-w_4_fos_skewness: The first-order statistics feature that describes the skewness value of the intensity levels in CET1-w images in X_{LHH} images.

$$skewness = \frac{\frac{1}{N}\sum_{i=1}^{N}(X(\mathrm{i})-\bar{X})^3}{\left(\sqrt{\frac{1}{N}\sum_{i=1}^{N}(X(\mathrm{i})-\bar{X})^2}\right)^3}$$

CET1-w_6_fos_skewness: The first-order statistics feature that describes the skewness value of the intensity levels in CET1-w images in X_{HLH} images. The formula is the same as has been shown for ***CET1-w_4_fos_skewness.***

CET1-w_1_GLCM_cluster_shade: The cluster shade in the gray-level co-occurrence matrix of textural features in CET1-w images in X_{LLL} images:

$$cluster\ shade = \sum_{i=1}^{N_g}\sum_{j=1}^{N_g}[i + j - \mu_x(i) - \mu_y(j)]^3\boldsymbol{P}(i,j)$$

T2-w_7_fos_mean: The first-order statistics feature that describes the mean value of the intensity levels in T2-w images in X_{HHL} images. The formula is the same as has been shown for ***CET1-w_4_fos_mean.***

T2-w_1_GLCM_cluster_shade: The cluster shade in the gray-level co-occurrence matrix of textural features in T2-w images in X_{LLL} images. The formula is the same as has been shown for ***CET1-w_1_GLCM_cluster_shade.***

T2-w_4_GLCM_cluster_shade: The cluster shade in the gray-level co-occurrence matrix of textural features in T2-w images in X_{LHH} images. The formula is the same as has been shown for ***CET1-w_1_GLCM_cluster_shade.***

T2-w_6_GLCM_autocorrelation: The autocorrelation in the gray-level co-occurrence matrix of textural features in T2-w images in X_{HLH} images.

$$autocorrelation = \sum_{i=1}^{N_g}\sum_{j=1}^{N_g} ij\boldsymbol{P}(i,j)$$

T2-w_6_GLCM_IMC1: Informational measure of correlation 1 in the GLCM in T2-w images in X_{HLH} images:

$$ICM1 = \frac{HXY2 - HXY1}{\max\{\mathrm{HX}, \mathrm{HY}\}}$$

Among there:

$HX = -\sum_{i=1}^{N_g} p_x(i)\log_2[p_x(i)]$ is the entropy of p_x;

$HY = -\sum_{i=1}^{N_g} p_y(i)\log_2[p_y(i)]$ is the entropy of p_y;

$$HXY1 = -\sum_{i=1}^{N_g}\sum_{j=1}^{N_g} P(i,j)\log(p_x(i)p_y(j))$$

$$HXY2 = -\sum_{i=1}^{N_g}\sum_{j=1}^{N_g} p_x(i)p_y(j)\log(p_x(i)p_y(j))$$

T2-w_7_GLCM_sum_average: The sum average in the gray-level co-occurrence matrix of textural features in T2-w images in X_{HHL} images.

$$sum\ average = \sum_{i=2}^{2N_g}[i\boldsymbol{P}_{x+y}(i)]$$

T2-w_1_GLRLM_LRHGLE: The long-run high gray level emphasis in the gray-level run-length matrix of textural features in X_{LLL} images:

$$LRHGLE = \frac{\sum_{i=1}^{N_g}\sum_{j=1}^{N_r}\left[\frac{p(i,j|\alpha)j^2}{i^2}\right]}{\sum_{i=1}^{N_g}\sum_{j=1}^{N_r} p(i,j|\alpha)}$$

***Methods 2*: Radiomic score (Rad-score) calculation formula**

Rad-score$_1$ =0.581*T1_3_fos_median

+0.138*T1_Surface_to_volume_ratio

+0.195 *T1_3_fos_mean

+0.987*T1_4_fos_mean

+-0.400*T1_4_fos_median

+0.385*T1_4_fos_skewness

+0.165*T1_6_fos_skewness

+-0.384*T1_1_GLCM_cluster_shade

Rad-score$_2$ = -0.3328*T2_7_fos_mean

+-0.4091*T2_1_GLCM_cluster_shade

+ 0.0723*T2_4_GLCM_cluster_shade

+ -0.1433 *T2_6_GLCM_autocorrelation

+ 0.3389*T2_6_GLCM_IMC1

+0.3946* T2_7_GLCM_sum_average

+ 0.1381*T2_1_GLRLM_LRHGLE

Rad-score = 0.88663*Rad-score$_1$

+0.50748* Rad-score$_2$

+0.02159*Gender

+0.02012*Age

+0.00691*HGB

-0.21954*N-stage

Reference

1. Haralick RM, Shanmugam K, Dinstein I. Textural Features for Image Classification. IEEE Transactions on Systems, Man, and Cybernetics. 1973; SMC-3: 610-21.
2. Chu A, Sehgal CM, Greenleaf JF. Use of gray value distribution of run lengths for texture analysis. Pattern Recognition Letters. 1990; 11: 415-9.
3. Galloway MM. Texture analysis using gray level run lengths. Computer Graphics and Image Processing. 1975; 4: 172-9.
4. van Griethuysen JJM, Fedorov A, Parmar C, Hosny A, Aucoin N, Narayan V, et al. Computational Radiomics System to Decode the Radiographic Phenotype. Cancer research. 2017; 77: e104-e7.
5. Yang Z, Zhuan B, Yan Y, Jiang S, Wang T. Identification of gene markers in the development of smoking-induced lung cancer. Gene. 2016; 576: 451-7.
6. Zhu J, Hastie T. Classification of gene microarrays by penalized logistic regression. Biostatistics (Oxford, England). 2004; 5: 427-43.
